# EMP1 regulates cell proliferation, migration and invasion in triple negative breast cancer through PI3K‐AKT signaling

**DOI:** 10.3389/fonc.2025.1701470

**Published:** 2025-12-11

**Authors:** Zheng Shu, Yonghao Li, Benshuo Zhu, Qinglin Zhou, Nuofan Li, Zike Shan, Mei Zhang

**Affiliations:** 1 Department of General Surgery, The First Affiliated Hospital of Shandong First Medical University & Shandong Provincial, Jinan, China; 2 Shandong First Medical University(Shandong Academy Of Medical Sciences), Jinan, China; 3 Shandong Second Medical University, Jinan, China

**Keywords:** EMP1, TNBC, Pi3k-akt, cancer, passway

## Abstract

**Introduction:**

Triple negative breast cancer (TNBC) is characterized by high malignancy and poor prognosis due to the lack of a clear therapeutic target. The search for therapeutic targets for TNBC has always been a focus of research in the field of human oncology. Existing studies have shown that Epithelial membrane protein 1 (EMP1) is abnormally expressed in a variety of cancers and is closely related to the occurrence and development of tumors. However, the potential role and molecular mechanism of EMP1 in TNBC are still unclear.

**Methods:**

In our study, we detected the expression levels of EMP1 in TNBC and analyzed the biological behavior of the TNBC cell line MDA-MB-231 after EMP1 expression changes.

**Results:**

The results showed that the expression level of EMP1 in TNBC was lower than that in normal tissues, and its expression level was related to T stage, lymph node metastasis, clinical stage and overall survival. In addition, overexpression of EMP1 inhibited the proliferation, migration and invasion of MDA-MB-231, while the proliferation, migration and invasion of MDA-MB-231 cells were enhanced after the expression of EMP1 decreased. EMP1 functions through the PI3K-AKT pathway.

**Discussion:**

In summary, our findings suggest that EMP1 plays a biological role as a tumor suppressor in TNBC.

## Introduction

Breast cancer is one of the most common malignancies worldwide. According to the latest data for 2024, it accounts for 6.9% of all cancer deaths and 11.6% of all new cancer cases worldwide (1). Data from 2020 shows that breast cancer has surpassed lung cancer in the highest number of new cases (2). TNBC is the most malignant type of breast cancer. Unlike other types of breast cancer, the treatment methods are relatively limited due to the lack of therapeutic targets such as estrogen receptor (ER), progesterone receptor (PR) and human epidermal growth factor receptor 2(HER2). Although the comprehensive treatment of breast cancer continues to develop, during clinical treatment, more than 90% of deaths in breast cancer patients are due to tumor metastasis (3, 4).

EMP1 belongs to the growth arrest-specific3 (GAS3)/peripheral myelin protein 22 kDa (PMP22) family, also called tumor-associated membrane protein (TMP) (5), progression-associated protein (PAP) (6), CL-20 (7), and was originally isolated from fetal rat intestine complementary DNA library and named by the Swiss scholar Taylor (8). EMP1 is expressed not only in normal tissues, but also aberrantly in malignant tumor tissues (9–13). Several studies have shown that EMP1 is involved in the biological behavior of tumor cells through tumor-associated signaling pathways (8, 14, 15). Currently, there are few studies on breast cancer on EMP1. Gnirke demonstrated a correlation between the expression of EMP1 and its aggressive and metastatic properties in several human breast cancer cell lines with different metastatic characteristics (16). Wang et al. found that tumor cell-derived EMP1 is crucial for the infiltration of cancer-associated fibroblasts in the tumor microenvironment of triple-negative breast cancer (17). Gulisa demonstrated that EMP1 can be used as a reliable marker to distinguish between the two most common histological types of breast cancer: invasive ductal carcinoma and lobular carcinoma, and that the mRNA expression of EMP1 is significantly higher in the latter (93.1%) than in the former (16.3%) (18). Because lobular carcinoma resists neoadjuvant therapy (18), the survival rate of patients with these tumors is lower than that of ductal carcinoma patients (19, 20), and these data may suggest that EMP1 has the function of promoting tumor progression. However, Sun found that EMP1 is underexpressed in breast cancer and may play an important role as a negative regulator of breast cancer MCF-7 cells by regulating the expression of caspase 9 and VEGFC proteins (9).Thus, future studies are required to explain the discrepancy of EMP1 functions in breast cancer.

Previous studies have not elucidated the biological mechanisms by which EMP1 influences the progression of TNBC or the signaling pathways involved. This study aims to investigate the expression of EMP1 in TNBC and its impact on biological behavior, further exploring the molecular pathways involved to clarify specific biological mechanisms. Delving into the underlying molecular mechanisms will not only aid in identifying patient groups at highest risk of recurrence but also provide new molecular targets for developing breast cancer treatment strategies.

## Materials and methods

### Bioinformatics

The GEPIA interactive web platform employs standardized pipelines to analyze RNA sequencing expression data derived from 9,736 tumor and 8,587 normal samples sourced from the TCGA and GTEx projects (21). The UALCAN database integrates TCGA datasets encompassing mRNA, miRNA, lncRNA, and DNA methylation profiles, enabling flexible exploration of relationships between tumor subgroups and clinical characteristics (22). First, EMP1 expression levels in breast cancer tissues versus normal tissues were analyzed using the GEPIA bioinformatics database. Second, EMP1 expression patterns across distinct breast cancer pathological subtypes were examined through the UALCAN database.

### Patients and tissue samples

Paired breast cancer tissues and adjacent non-tumorous tissues were collected from 60 TNBC patients at the First Affiliated Hospital of Shandong First Medical University. Immediately after surgical resection, all specimens were snap-frozen in liquid nitrogen. Histopathological examination confirmed that all specimens were diagnosed with TNBC without preoperative treatment. Clinicopathological characteristics of the cohort are summarized in **Table 1**. This study was approved by the Institutional Ethics Committee of the First Affiliated Hospital of Shandong First Medical University and conducted in accordance with the Declaration of Helsinki(S414). Informed consent was obtained from all subjects and/or their legal guardians in our study.

**Table 1:** Basic pathological information of patients.

Clinicopathological characteristics	No.	EMP1 expression level		P value (Chi-square test)
		Low	High	
Age				
≤50	27	12	15	0.436
>50	33	18	15	
T stage				
T1	14	12	2	0.016
T2	15	8	7	
T3	11	6	5	
T4	20	6	14	
N stage				
N0	17	14	3	0.040
N1	12	8	4	
N2	12	6	6	
N3	19	7	12	
Clinical staging				
I	16	10	6	0.0009
II	16	8	8	
III	10	4	6	
IV	18	0	18	
Distant metastasis				
Yes	24	10	14	0.264
No	36	10	26	

### Cell culture

Purchased the TNBC cell line MDA-MB-231 from Wuhan Pronoxy Life Science Company. In this study, the TNBC cell line MDA-MB-231 was cultured in DMEM supplemented with 10% fetal bovine serum (FBS) and 1% penicillin-streptomycin antibiotic cocktail. Cells were maintained at 37 °C in a humidified 5% CO_2_ incubator. Cell growth status was monitored regularly to assess potential contamination. Cells in the logarithmic growth phase with optimal viability were selected through routine passaging for subsequent experiments.

### Cell transfection

Cells were seeded into 6-well plates at a density of 1 × 10^5^ cells per well, with three replicates per experimental group. Following incubation, cell growth was monitored until 70-80% confluence was achieved. For transfection, the culture medium was aspirated and cells were washed twice with phosphate-buffered saline (PBS) for 5 min per wash. The plasmid DNA and short hairpin RNA (shRNA) were purchased from GENERAL BIOL, and the transfection performed was transient. Plasmid DNA or short hairpin RNA (shRNA) was complexed with Lipofectamine 3000 transfection reagent at a 1:50 ratio in Opti-MEM reduced-serum medium. The mixture was incubated at room temperature for 20 min before being added dropwise to the cells. Transfected cells were maintained in serum-free medium for 4–6 h, followed by replacement with complete growth medium. RNA and protein were harvested 24–72 h post-transfection for efficiency validation.

### Real-time fluorescent quantitative PCR

Frozen tumor and adjacent tissues stored at -80°C were retrieved from liquid nitrogen. For RNA extraction, tissues (50–100 mg) were homogenized in 1 ml Vazyme VE-ZOL Reagent (Vazyme Biotech, Nanjing, China) using glass homogenizers. Total RNA was quantified with a NanoPhotometer spectrophotometer. The purified RNA was subsequently used for reverse transcription and quantitative real-time PCR (qRT-PCR). For cell line analysis, RNA was extracted 24 hours post-transfection using the aforementioned rapid extraction kit following cell harvesting and trypsinization. Relative RNA expression levels were calculated using the comparative 2^(-ΔΔCt) method, with normalization to housekeeping genes. The primer sequences were as follows: EMP1 forward: 5’-TACCTGGACGAGATTCCCCC-3’ and reverse 5’-TCTCAACTCCCCCAGTTCCT-3’.β-Actin forward: 5’-GAGAAAATCTGGCACCACACC-3’ and reverse 5’-GATAGCACAGCCTGGATAGCAA-3’.

### Western blot assay

Forty-eight hours post-transfection, cells were trypsinized, collected, and transferred to 1.5 mL microcentrifuge tubes. Ice-cold RIPA lysis buffer was added for protein extraction. Following quantification of protein concentration, 60 μg of total protein per sample was resolved by 10% SDS-PAGE and electrophoretically transferred to PVDF membranes at 300 mA for 60 min. Membranes were blocked with 5% non-fat dry milk in TBST for 90 min at room temperature, then incubated overnight at 4 °C with primary antibodies against: EMP1 (ABCAM, ab230445, 1:1,000), PI3K (proteintech, 30092-1-AP, 1:1,000), phospho-PI3K (ABSIN, abs130868, 1:500), AKT (proteintech, 10176-2-AP, 1:2,000), phospho-AKT (proteintech, 80462-1-RR, 1:2,000), and GAPDH (ABCAM, ab9485, 1:2,500). After three 10-min TBST washes, membranes were incubated with horseradish peroxidase-conjugated secondary antibody (HRP-conjugated Goat Anti-Rabbit IgG(H+L), proteintech, SA00001-2, 1:10,000) for 2 h at room temperature. Protein bands were visualized using enhanced chemiluminescence substrate.

### Cell counting kit-8 assay

MDA-MB-231 cell suspensions were seeded in 96-well plates at a density of 5×10^4^ cells/mL (100 μL per well), with five replicate wells per experimental group (overexpression vs. control). Peripheral wells were filled with phosphate-buffered saline (PBS) to minimize evaporation. Following 24-hour pre-culture in a humidified 37 °C/5% CO_2_ incubator to ensure cell adherence, 10 μL of CCK-8 solution was added to each well. After 2-hour incubation at 37 °C, absorbance at 450 nm was measured using a microplate reader. Measurements were taken at 0, 24, 48 and 72-hour timepoints post-seeding. Cell viability was calculated using the following formula:

Cell viability (%) = [(As - Ab)/(Ac - Ab)] × 100%.

where As represents the absorbance of the experimental well (containing cells, CCK-8 reagent, and treatment), Ac represents the absorbance of the control well (containing cells and CCK-8 reagent without treatment), and Ab represents the absorbance of the blank well (containing culture medium and CCK-8 reagent without cells). All experiments were performed in triplicate (n = 3 biological replicates).

### Transwell invasion assay

Transfected cells were obtained and resuspended in serum-free medium at a density of 5*10^6^ cells/mL. For invasion assays, 500 μL of medium containing 10% fetal bovine serum (FBS) was added to the lower chamber of a 24-well plate. Subsequently, 200 μL of cell suspension was seeded into the upper chamber of Matrigel-coated Transwell inserts (8 μm pore size; Corning, USA). After 48-hour incubation at 37 °C/5% CO_2_, non-invading cells on the upper membrane surface were removed with cotton swabs. Invaded cells on the lower surface were fixed with 4% paraformaldehyde for 15 min and stained with 0.1% crystal violet for 20 min. Microscopic images were acquired at a magnification of ×200 using an Olympus IX83 microscope. For each replicate within an experimental group (n=3 biological replicates), three fields of view were randomly selected and captured in a blinded manner to avoid bias. Cell counting was performed using ImageJ software (NIH, USA). Statistical counting was performed after 3 parallel experiments.

### Wound healing assay

Cell migration was assessed using a wound healing assay. MDA-MB-231 cells were seeded in 6-well plates at 5×10^5^ cells/well and cultured overnight in complete medium at 37 °C with 5% CO_2_ until >90% confluence. Uniform wounds were created by scraping the monolayer with a sterile P200 pipette tip. After washing with PBS to remove detached cells, cells were maintained in medium containing 1% FBS. Wound closure was monitored at 0, 24, and 48 h using a phase-contrast microscope (Nikon Eclipse TS2, 40×). The migratory capacity was quantified by measuring residual wound area with ImageJ software (v1.53; NIH, USA) and expressed as percentage closure:


$$\text{Closure }\left(\%\right)\;=\;\left[1\;-\;\left({\text{Wound area}}_{\text{t}}\;/{\text{ Wound area}}_{\text{o}}\right)\right]\;\times \;100.$$

All experiments were performed in triplicate (n = 3 biological replicates).

### Animal experiment

Female, 4-week-old BALB/c nude mice were purchased from Jinan Pengyue Laboratory Animal Company. Ten 5- to 6-week-old female BALB/c athymic nude mice (body weight: 16–20 g) were randomly assigned to two groups (n=5/group). Mice were maintained under specific pathogen-free conditions with ad libitum access to food and water. Before implanting tumors under the skin, mice were given intraperitoneal injection anesthesia, and ketamine was diluted to 10 mg/mL with sterile saline and toluethazide to 1 mg/mL. Mix by volume ratio 1:1. Intraperitoneal injection at a dose of 0.1 mL/10 g body weight. Cells transfected with EMP1-expression plasmids(Oe1-EMP1) or empty vector (2×10^5^ cells in 100 μL PBS per injection) were subcutaneously inoculated into the right flank of each mouse. Tumor dimensions were measured weekly using digital calipers, with volume (V) calculated as:


$$\text{V }=\;\left({\text{Width}}^{2}\;\times \text{ Length}\right)/2$$

At 4 weeks post-inoculation, mice were euthanized by CO_2_ asphyxiation. Tumors were surgically excised, photographed, and weighed. After tumor removal, RNA and proteins were extracted. Transfection efficiency was assessed via qRT-PCR and Western Blot to confirm changes in EMP1 levels following *in vivo* xenografting (**Supplementary Figure 1**). All procedures were approved by the Institutional Animal Care and Use Committee of Shandong First Medical University (No: S670). We confirm that all experiments are conducted in accordance with relevant guidelines and regulations. We confirm that the study is reported in accordance with ARRIVE guidelines.

### Survival prognosis analysis

To evaluate the correlation between EMP1 expression and overall survival (OS) in patients, gene expression data and corresponding clinical survival data were selected from the triple-negative breast cancer cohort in the Cancer Genome Atlas (TCGA) database. EMP1 expression levels were quantified using probe set 213895_at on the Affymetrix HT-HG-U133A chip. Probe-level expression values underwent log2(signal value + 1) transformation to approximate a normal distribution. Patients were stratified into high-expression and low-expression groups based on the median EMP1 expression value. Survival curves were plotted using the Kaplan-Meier method and compared via the Log-rank test. Univariate Cox proportional hazards regression models were employed to calculate hazard ratios (HR) and their 95% confidence intervals (CI). Patient survival rates were recorded at key follow-up time points (0, 50, 100, 150, and 200 months). A p-value < 0.05 was considered statistically significant.

### Statistical analysis

Statistical analyses were performed using IBM SPSS Statistics 26.0. Quantitative data with normal distribution were analyzed by Student’s *t*-test (two-group comparisons) or one-way ANOVA (multi-group comparisons), while categorical variables were assessed using chi-square tests. Significance thresholds were defined as *p < 0.05,**p < 0.01, ***p < 0.001, ****p < 0.0001. All experiments included three independent replicates.

## Results

### Low expression of EMP1 in TNBC

**Figure 1:**
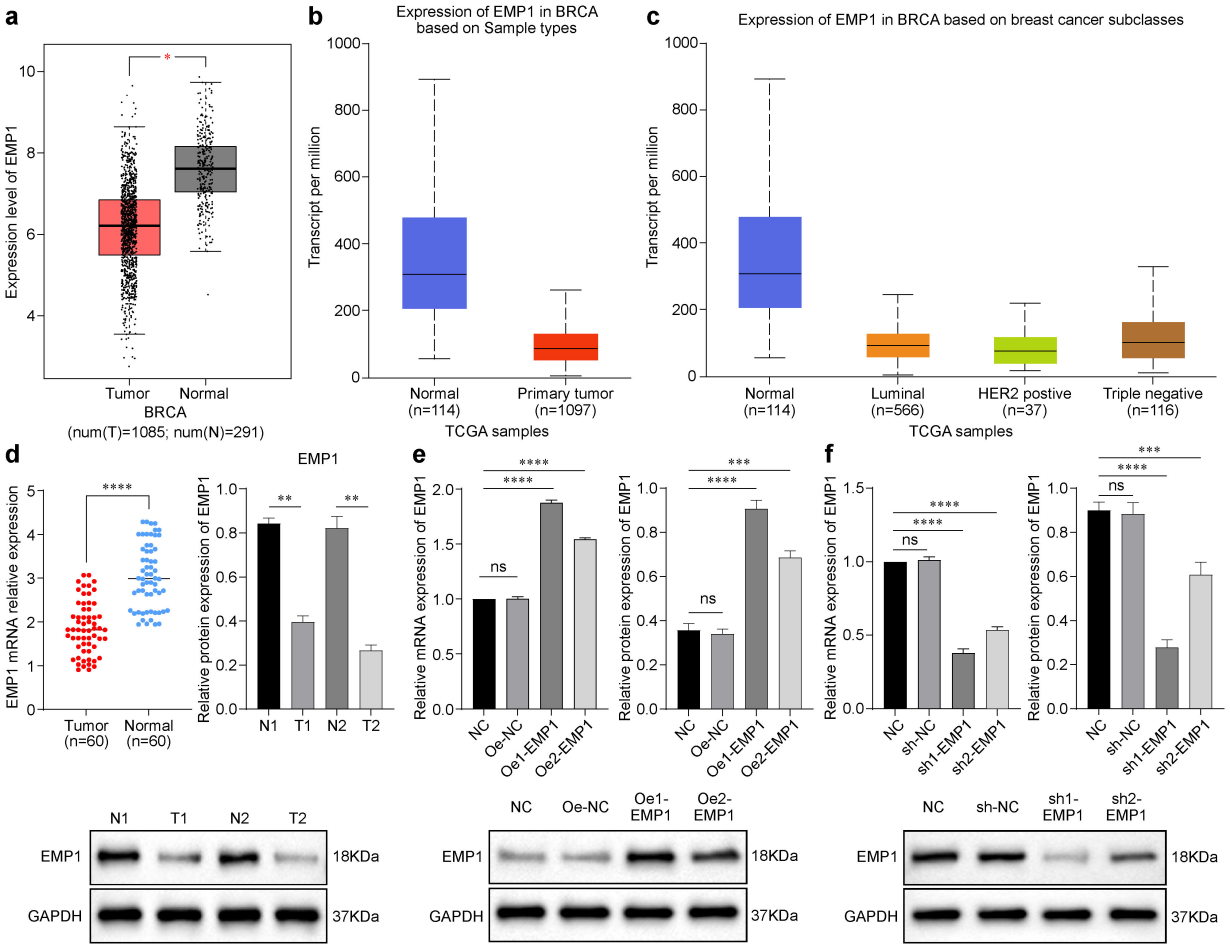
EMP1 expression in breast cancer via bioinformatic analysis and experimental validation and Validation of EMP1 knockdown and overexpression **(a)** EMP1 mRNA expression in breast cancer (BRCA) vs. normal tissues from GEPIA database (p < 0.05); **(b)** EMP1 mRNA expression in breast cancer (BRCA) vs. normal tissues from UALCAN database (p < 0.001); **(c)** EMP1 expression across BRCA molecular subtypes (Luminal, HER2+, TNBC) via UALCAN; **(d)** qRT-PCR analysis of EMP1 mRNA in 60 paired TNBC and adjacent normal tissues (p < 0.0001) and Western blot and quantitative analysis of EMP1 protein in two representative TNBC cases vs. normal tissues (p < 0.05); **(e)** The efficiency of EMP1 overexpression was verified using qRT-PCR and Western Blot (Oe vs. Oe-NC); **(f)** The efficiency of EMP1 knockdown was verified using qRT-PCR and Western Blot (sh vs. sh-NC). (GAPDH loading control shown. Data: mean ± SD. *p < 0.05, **p < 0.01, ***p < 0.001, ****p < 0.0001)(NC: Negative Control; Oe: Overexpression; Oe-NC: empty vector; sh:Short Hairpin).

The GEPIA database analysis revealed significantly reduced EMP1 expression in breast cancer tissues compared to normal tissues (**Figure 1a**). Consistent with this, UALCAN data demonstrated low EMP1 levels across all major molecular subtypes (Luminal, HER2+, and TNBC) of breast cancer (**Figure 1b, c**). Based on these findings, we specifically quantified EMP1 mRNA in TNBC using qRT-PCR. Analysis of 60 paired TNBC and adjacent normal tissues showed marked downregulation of EMP1 mRNA in tumors (**Figure 1d**). Finally, Western blot analysis of 60 TNBC tissues validated significantly reduced EMP1 protein expression compared to normal counterparts (**Figure 1d**).

### Construction of TNBC cells with stable overexpression or knockdown of EMP1

Transfection efficiency was rigorously validated through qRT-PCR and Western blotting. EMP1 overexpression (Oe1, Oe2) significantly elevated mRNA levels versus control (Oe-NC), concordantly enhancing protein expression (**Figure 1e**). Conversely, EMP1 knockdown (sh1, sh2) reduced mRNA and protein levels relative to sh-NC (**Figure 1f**).

### Overexpression of EMP1 inhibits proliferation, migration, and invasion of MDA-MB-231 TNBC cells *in vitro* and *in vivo*

**Figure 2:**
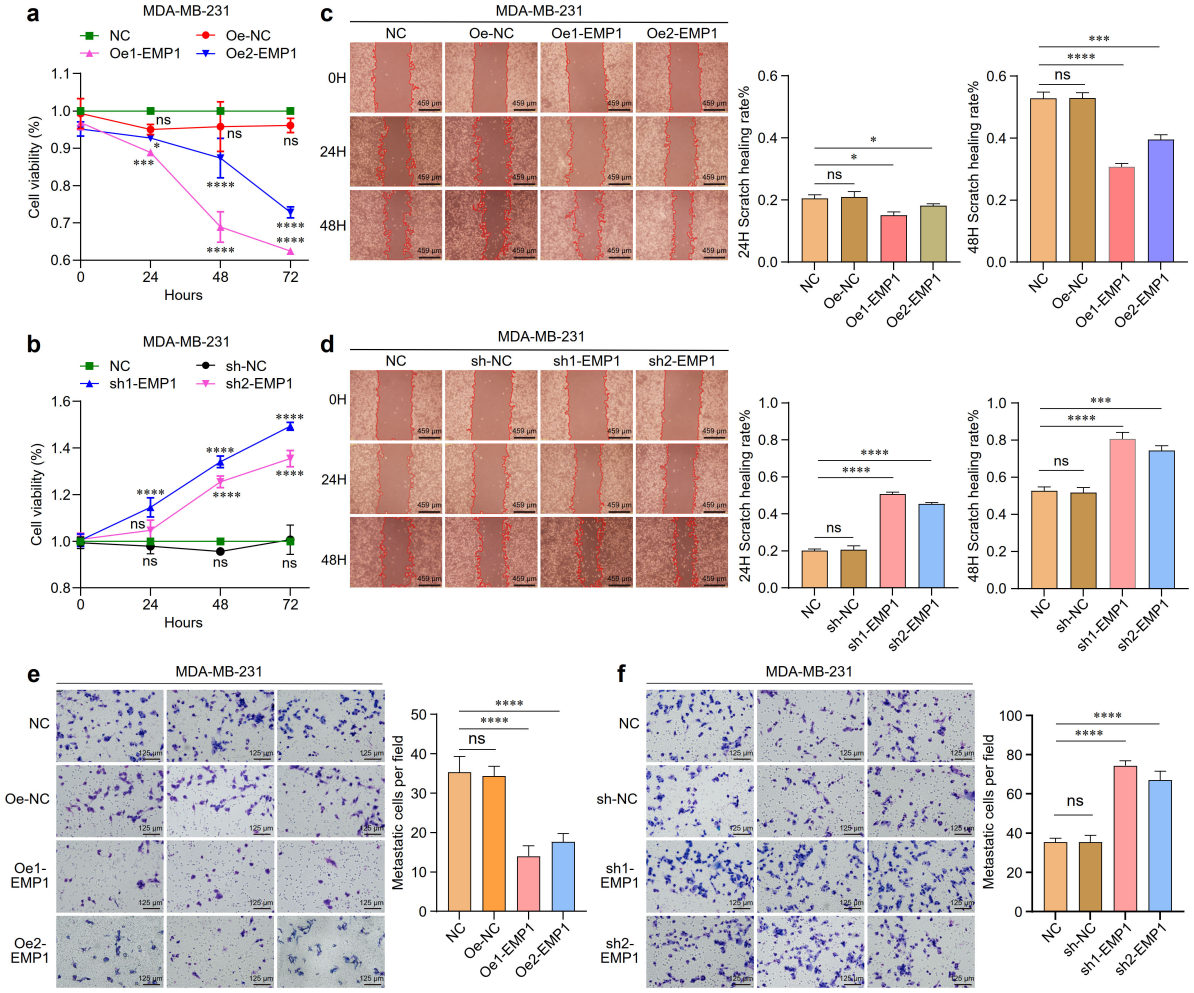
The effect of EMP1 on the proliferation, migration, and invasion of MDA-MB-231 TNBC cells *in vitro*. **(a)** Proliferation of MDA-MB-231 cells with EMP1 overexpression was measured by CCK-8 assay at 0, 24, 48, and 72 hours; **(b)** Proliferation of MDA-MB-231 cells with EMP1 knockdown was measured by CCK-8 assay at 0, 24, 48, and 72 hours; **(c)** Cell migration ability evaluated by wound healing assay in EMP1-overexpressing MDA-MB-231 cells. Representative images at 24h and 48h are shown; **(d)** Cell migration ability evaluated by wound healing assay in EMP1-knockdown MDA-MB-231 cells. Representative images at 24h and 48h are shown; **(e)** Cell invasion capacity determined by Transwell invasion assay in EMP1-overexpressing MDA-MB-231 cells; **(f)** Cell invasion capacity determined by Transwell invasion assay in EMP1-knockdown MDA-MB-231 cells. Scale bar: 459μm(c,d),125μm(e,f). (*p < 0.05, ***p < 0.001, ****p < 0.0001.) EMP1 overexpression suppresses tumor growth *in vivo*.

Cell proliferation was assessed using Cell Counting Kit-8 (CCK-8) assay. Consistent with a tumor suppressor role, EMP1 overexpression significantly inhibited proliferation, whereas EMP1 knockdown enhanced proliferative capacity (**Figures 2a, b**). Cell migration capacity was monitored by wound healing assays. EMP1 overexpression significantly inhibited cell migration, while EMP1 reduced expression enhanced cell migration capacity (**Figures 2c, d**). Cell invasive potential was evaluated using Matrigel-coated Transwell assays. EMP1 overexpression significantly hindered cellular invasion through the extracellular matrix, while decreased EMP1 expression facilitated aggressive penetration (**Figures 2e, f**).

**Figure 3:**
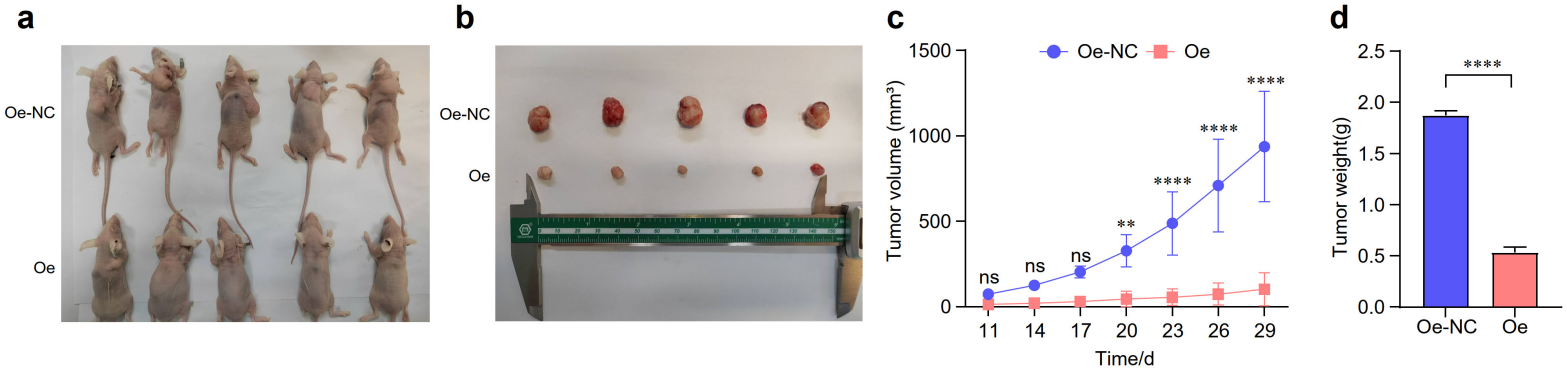
EMP1 suppresses tumor growth in vivo**. (a, b)** Representative photographs of nude mice and the resected tumors from the Oe-NC (empty vector control) and Oe-EMP1 groups (n=5 per group); **(c)** Tumor growth curves of xenograft tumors in each group. Data are presented as mean ± SD (n=5; **p<0.01; ****p<0.0001, two-way ANOVA); **(d)** Final tumor weights measured at the end of the experiment. Data are presented as mean ± SD (n=5;, p < 0.01, Student’s t-test).

Building on our *in vitro* findings, we validated the tumor-suppressive role of EMP1 in TNBC using an *in vivo* xenograft model. EMP1 overexpression robustly curbed tumor growth, as evidenced by reduced tumor volume and weight compared to control groups (**Figures 3a–d**).

### EMP1 regulated the PI3K/AKT signaling pathway in MDA-MB-231 TNBC cells

To explore the molecular mechanism by which EMP1 modulates triple-negative breast cancer (TNBC) progression, we probed the PI3K/AKT pathway—a key regulator of TNBC pathogenesis.

**Figure 4:**
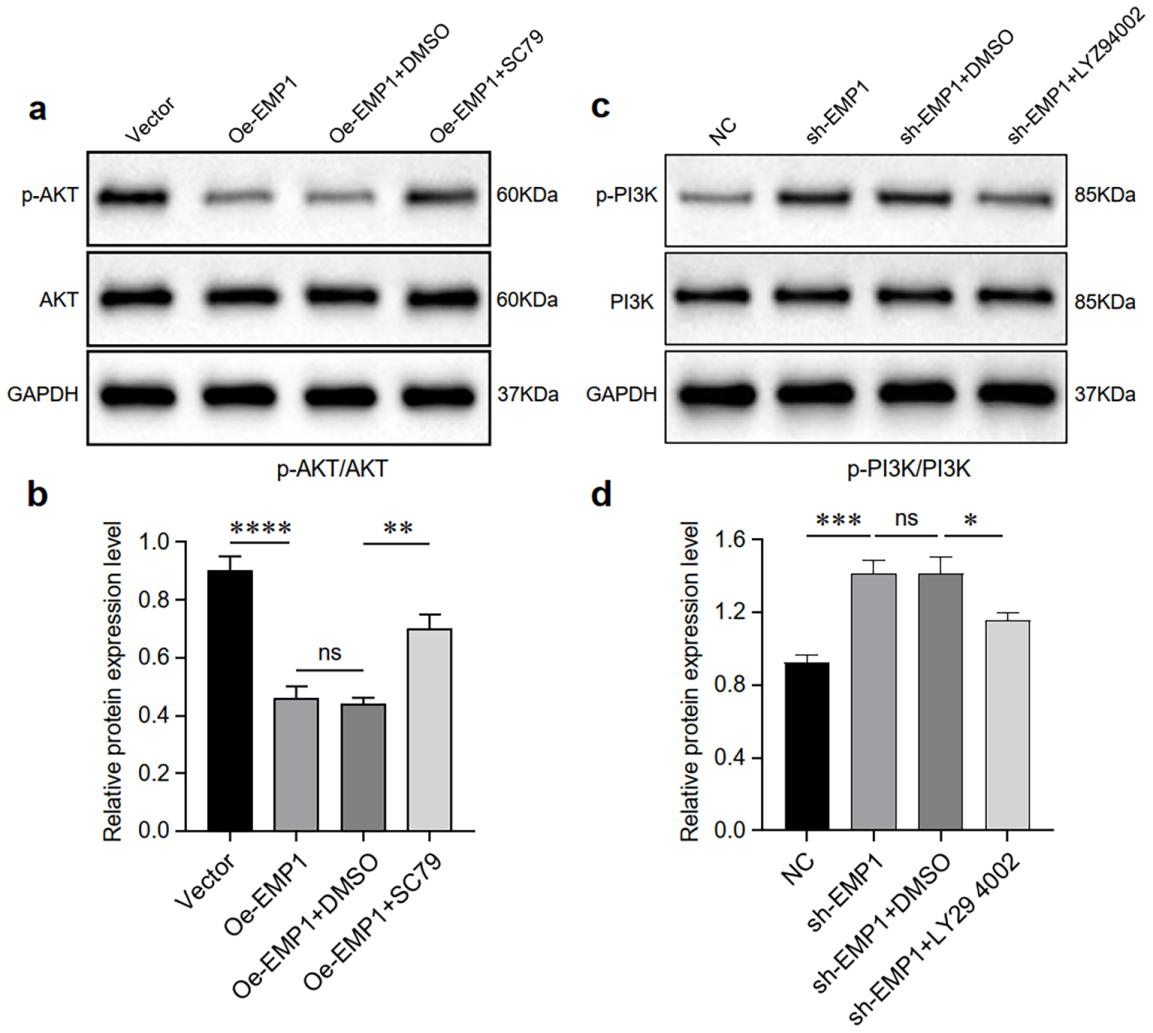
EMP1 suppresses the PI3K/AKT signaling pathway *in vitro*. **(a)** Western blot analysis of AKT and phosphorylated AKT (p-AKT) in EMP1-overexpressing cells treated with the AKT agonist SC79 or solvent control (DMSO); **(b)** Quantitative analysis of the p-AKT/AKT ratio. Data from three independent experiments are shown as mean ± SD (p < 0.01; NS, not significant; one-way ANOVA); **(c)** Western blot analysis of PI3K and phosphorylated PI3K (p-PI3K) in EMP1-knockdown cells treated with the PI3K inhibitor LY294002 or DMSO; **(d)** Quantitative analysis of the p-PI3K/PI3K ratio. Data from three independent experiments are presented as mean ± SD (*p < 0.05, **p < 0.01, ***p < 0.001, ****p < 0.0001; NS, not significant; one-way ANOVA).

As expected, EMP1 overexpression significantly downregulated the levels of phosphorylated AKT in MDA-MB-231 triple-negative breast cancer cells. However, the levels of total AKT did not undergo significant changes. Downregulation of EMP1 expression increased the levels of phosphorylated PI3K in MDA-MB-231 triple-negative breast cancer cells. To enhance experimental credibility, we established AKT activator SC79 and PI3K inhibitor LY294002, along with their respective control groups (DMSO). Results indicate that the AKT activator SC79 partially counteracted the effects of EMP1 overexpression (**Figures 4a, b**), while the PI3K inhibitor LY294002 partially reversed the effects of reduced EMP1 expression (**Figures 4c, d**).

### Low expression of EMP1 suggests a poor prognosis

**Figure 5:**
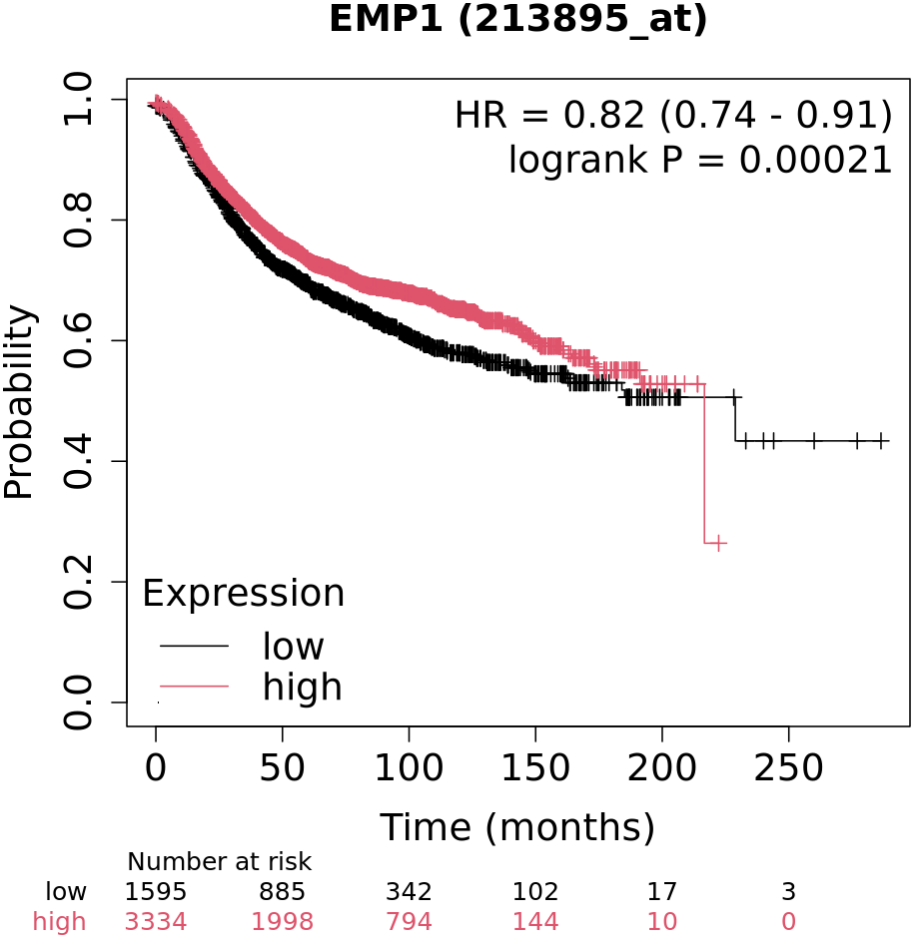
Association of EMP1 expression with overall survival in TNBC.

Survival analysis results indicate that EMP1 expression levels are significantly correlated with overall survival in patients. As shown in **Figure 5**, patients in the high-expression group demonstrated superior survival outcomes compared to those in the low-expression group (Log-rank P = 0.00021). Univariate Cox regression analysis further confirmed that high EMP1 expression is a significant protective factor (HR = 0.82, 95% CI: 0.74–0.91, Log-rank P = 0.00021).

Patients were stratified into high and low expression groups based on the median expression level of EMP1. EMP1 High and EMP1 Low groups are shown in red and black, respectively. The Log-rank test was used to calculate the P-value. HR, hazard ratio; CI, confidence interval.

## Discussion

Breast cancer represents the second leading cause of cancer-related mortality among women worldwide (23). While early-stage disease is generally considered curable, advanced breast cancer with distant metastases remains incurable (24). Significant morphological and molecular heterogeneity characterizes this malignancy, necessitating individualized therapeutic strategies based on molecular subtypes, which directly influence clinical prognosis. Current research prioritizes advanced molecular detection technologies to dissect this heterogeneity, enabling earlier diagnosis and improved survival outcomes (25). Through integrated *in vivo* and *in vitro* investigations, we demonstrate that EMP1 expression is downregulated in TNBC. Mechanistically, EMP1 suppresses tumor progression by inhibiting cell proliferation, invasion, and migration through modulation of the PI3K/AKT signaling pathway.

EMP1 exhibits paradoxical roles in tumorigenesis and cancer progression, manifesting not only across diverse malignancies but also in distinct cellular behaviors. In colorectal cancer, oral squamous cell carcinoma, and laryngeal carcinoma, EMP1 functions as a tumor suppressor by inhibiting proliferation and inducing apoptosis (9, 26, 27). Conversely, it acts as an oncogenic promoter in glioblastoma and bladder urothelial carcinoma, enhancing migration and invasion (11, 28). This functional duality is closely associated with context-dependent factors including the tumor microenvironment, genetic landscape, and activation states of specific signaling pathways. Deeper mechanistic insights into EMP1’s divergent functions could inform targeted therapeutic strategies. Although seminal work by Gnirke et al., Gulisa et al., and Sun et al. has established EMP1’s critical involvement in breast cancer pathogenesis (9, 16–18), its precise mechanistic role in TNBC remains to be elucidated.

The PI3K/AKT signaling pathway critically regulates tumorigenesis. EMP1 overexpression in lung cancer enhances proliferation through PI3K/AKT activation (29). Similarly, MicroRNA-95-3p-mediated targeting of EMP1/PI3K/AKT signaling confers cisplatin resistance in gastric cancer (30). Given PI3K/AKT’s established roles in proliferation and migration, we quantified pathway activity in TNBC cells. Our data demonstrate that EMP1 functions as an upstream regulator of PI3K/AKT signaling. Elucidating EMP1’s precise mechanistic actions through this pathway offers critical implications for therapeutic development. Current evidence suggests EMP1 may suppress PI3K activation either directly or indirectly via (31): (i) Modulation of RTK membrane localization/stability; (ii) Alteration of upstream effector activity; (iii) Interactions with membrane protein complexes. While EMP1’s involvement in TNBC pathogenesis is recognized, its exact molecular mechanisms remain incompletely characterized. Identification of EMP1-interacting partners and signaling dynamics constitutes a priority for future investigations.

Despite rigorous experimental design, this study has inherent limitations. First, EMP1 expression validation was restricted to 60 paired tissue specimens; future research should expand to larger multicenter cohorts. Second, the investigation of triple-negative breast cancer cells was restricted to a single cell line; further validation using other cell lines is required to exclude cell line specificity. Thirdly, due to time constraints, the subcutaneous tumor formation experiment in animals only included an overexpression group to verify whether EMP1 exerts tumor-suppressing effects, without establishing a knockdown group for further validation. Additionally, while this study confirmed that EMP1 function depends on the PI3K/AKT signaling pathway, the precise molecular mechanisms regulating this axis remain to be elucidated.

Collectively, we provide first evidence that EMP1 is downregulated in triple-negative breast cancer and suppresses malignant progression via the PI3K/AKT pathway.

## Data Availability

The datasets presented in this study can be found in online repositories. The names of the repository/repositories and accession number(s) can be found in the article/**Supplementary Material**.

## Supplementary material

Supplementary Figure 1 EMP1 overexpression is maintained *in vivo.***(a)** qRT-PCR analysis of human EMP1 mRNA levels in xenograft tumors from mice injected with vector control or EMP1-overexpressing (Oe-EMP1) cells. **(b)** Representative Western blot analysis of EMP1 protein expression in the resected tumors. **(c)** Quantitative analysis of EMP1 protein expression from **(b)**, normalized to GAPDH. All data are shown as mean ± SD. ***p < 0.001, **p < 0.01, *p < 0.05.
