# Supplementary figures and images for: EMP1 regulates cell proliferation, migration and invasion in triple negative breast cancer through PI3K‐AKT signaling
Source: Front Oncol. 2025 Dec 11;15:1701470. doi: 10.3389/fonc.2025.1701470 (PMC12738301; doi:10.3389/fonc.2025.1701470)

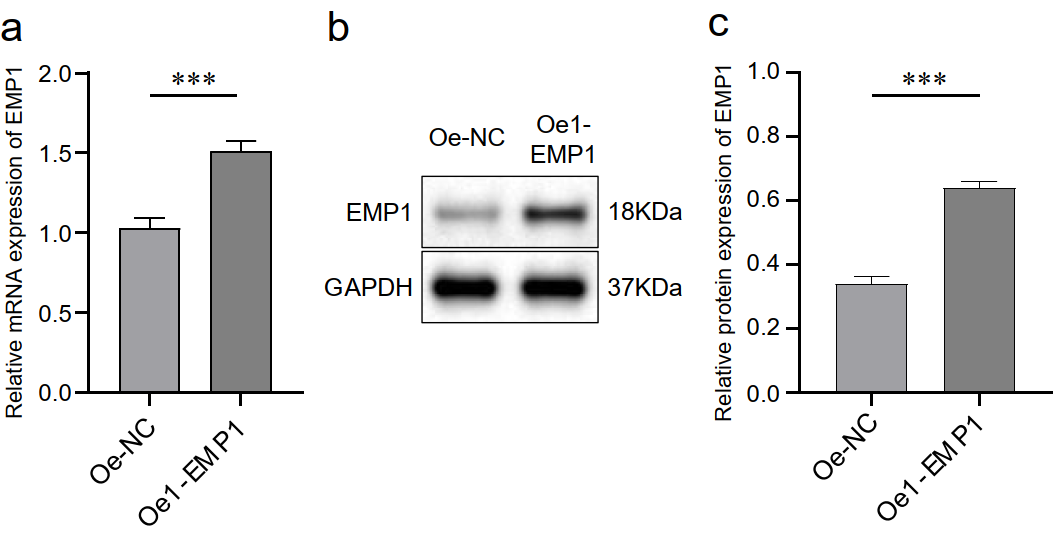

Supplement: Supplementary Figure 1 — EMP1 overexpression is maintained in vivo.(a) qRT-PCR analysis of human EMP1 mRNA levels in xenograft tumors from mice injected with vector control or EMP1-overexpressing (Oe-EMP1) cells. (b) Representative Western blot analysis of EMP1 protein expression in the resected tumors. (c) Quantitative analysis of EMP1 protein expression from (b), normalized to GAPDH. All data are shown as mean ± SD. ***p < 0.001, **p < 0.01, *p < 0.05. [file Image1.tif]
